# Ensemble Learning Framework with GLCM Texture Extraction for Early Detection of Lung Cancer on CT Images

**DOI:** 10.1155/2022/2733965

**Published:** 2022-06-02

**Authors:** Sara A. Althubiti, Sanchita Paul, Rajanikanta Mohanty, Sachi Nandan Mohanty, Fayadh Alenezi, Kemal Polat

**Affiliations:** ^1^Department of Computer Science, College of Computer and Information Sciences, Majmaah University, Al-Majmaah, Saudi Arabia; ^2^Department of Computer Science & Engineering, Birla Institute of Technology, Mesra, Ranchi, India; ^3^Department of Computer Science & Engineering, Specialisation Program, Faculty of Engineering and /Technology, Jain University, Bangalore, India; ^4^Department of Computer Science & Engineering, Vardhaman College of Engineering (Autonomous), Hyderabad, India; ^5^Department of Electrical Engineering, College of Engineering, Jouf University, Saudi Arabia; ^6^Department of Electrical and Electronics Engineering, Bolu Abant Izzet Baysal University, Faculty of Engineering, Bolu, Turkey

## Abstract

Lung cancer has emerged as a major cause of death among all demographics worldwide, largely caused by a proliferation of smoking habits. However, early detection and diagnosis of lung cancer through technological improvements can save the lives of millions of individuals affected globally. Computerized tomography (CT) scan imaging is a proven and popular technique in the medical field, but diagnosing cancer with only CT scans is a difficult task even for doctors and experts. This is why computer-assisted diagnosis has revolutionized disease diagnosis, especially cancer detection. This study looks at 20 CT scan images of lungs. In a preprocessing step, we chose the best filter to be applied to medical CT images between median, Gaussian, 2D convolution, and mean. From there, it was established that the median filter is the most appropriate. Next, we improved image contrast by applying adaptive histogram equalization. Finally, the preprocessed image with better quality is subjected to two optimization algorithms, fuzzy c-means and k-means clustering. The performance of these algorithms was then compared. Fuzzy c-means showed the highest accuracy of 98%. The feature was extracted using Gray Level Cooccurrence Matrix (GLCM). In classification, a comparison between three algorithms—bagging, gradient boosting, and ensemble (SVM, MLPNN, DT, logistic regression, and KNN)—was performed. Gradient boosting performed the best among these three, having an accuracy of 90.9%.

## 1. Introduction

Carcinoma is the leading cause of death in the world. Carcinomas are cancers that start in cells that make up the skin or the tissue lining organs, such as the lungs or kidneys. Lung cancer, also known as carcinoma of the lungs, is characterized by an unrestricted growth of cells in lung tissue and distinguished by a specific growth pattern. Lung cancer is dangerous to leave untreated, as it may propagate to other body parts. Small-cell lung carcinoma and nonsmall-cell lung carcinoma are the two major categories, and the primary cause is smoking. Lung cancer has also been found in people with no smoking history but with exposure to air pollution, secondary smoking, and sometimes toxic gasses. Before the 12^th^ century, occurrence of lung cancer was actually very rare. But nowadays, it is widespread. Many patients consult a doctor only when their disease and symptoms become extreme, thereby making these disease and symptoms very difficult to diagnose and cure. Thus, early-stage treatment of lung cancer is crucial in saving lives. One way to detect the distinctive abnormal growth of cells is through X-ray. Another method of cancer detection is sputum cytology. If the lungs produce sputum, cancer can be seen by looking at the sputum through a microscope. Tissue sampling, also called biopsy, is another method for early detection of lung cancer. The conventional and most widespread method of detecting lung cancer is by using computer tomography (CT) and radiographs. CT scan uses X-ray and a computer to deliver a clear image of the lungs, giving better results than an X-ray alone. The CT scan image gives much more detail than a plain image, and the doctors can view a particular organ from different angles [1–33]. In this study, 20 lung image samples are taken for analysis. The image is denoised; then, the image is enhanced. Afterwards, features are extracted using GLCM. Lastly, classification is done. Integration of median filter, adaptive histogram equalization, and fuzzy c-means clustering for segmentation showed more accurate results. After applying feature extraction using GLCM (Haralick features), the accuracy of the ensemble classifier consisting of MLPNN, DT, SVM, and KNN classifiers was computed and confirmed to be highly effective. Thus, the study has great potential to advance the early detection of lung cancer.

## 2. Related Works

Senthil Kumar et al. [34] used a segmentation algorithm (k-means) on computer tomography (CT) scan images to detect lung cancer. Image segmentation was achieved by applying fuzzy c-means and k-means algorithms. Fuzzy c-means delivered enhanced performance in comparison to k-means. Using guaranteed convergence particle swarm optimization (GCPSO), an accuracy of 95.89% was achieved for the detection of lung cancer. Using a novel Multicrop Convolutional Neural Network (MC-CNN), an accuracy of 86.24% was achieved in identifying the lung module malignancy. In MC-CNN, features are extracted from the nodules by trimming distinct areas from convolution feature maps and applying max-pooling several times [35]. Sensitivity of 70%-90% was achieved using random forest and principal component analysis by extracting features using local shape analysis [36]. Using two successive k-nearest neighbor classifiers, a sensitivity of 80% was achieved using the curvedness and shape feature of the local image [37]. Accuracy of 95.91% was achieved using a probabilistic neural network (PNN) by extracting lung volume, and reduction was done using principal component analysis (PCA) [38]. Accuracy of 95.62% was achieved using texture, volumetric, intensity, and geometric features, and Fuzzy Particle Swarm Optimization (FPSO) was used for feature selection, with deep learning being applied for classification [39]. Sensitivity of 93.02% was achieved in detection detecting ground-glass opacity (GGO) using Support Vector Machine (SVM) twice and using four 2-dimensional features and 11 3-dimensional features [40]. Classification accuracy of 96% was achieved using speed up robust feature (SURF) along with genetic algorithms (GA) for optimization and a neural network (NN) for classification [41]. 97.61% accuracy was achieved using a genetic algorithm with wrapper approach (GAWA) using a multilevel brightness-preserving approach and segmentation using a deep neural network. Features are derived from the segment and selected using a generalized rough set (hybrid spiral optimization intelligent) [42]. An accuracy of 89.29% was obtained using two 3D deep learning models [43]. Using 2D and 3D shape and texture features and histogram, k-means clustering (autocenter) provided a sensitivity of 88.88%. [44]. Using volumetric CT data, sensitivity reached more than 90% using a 3D convolution neural network. [45–52].

## 3. Materials and Methods

**Figure 1:**
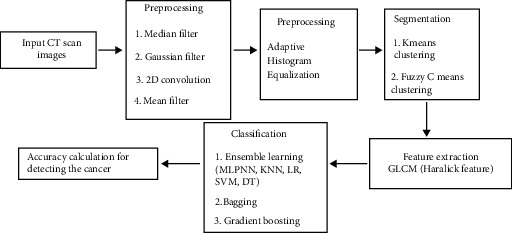
Block diagram of framework for detection of lung cancer.

Firstly, a filtering technique is used to filter out the noise from the 20 images. In this study, 4 filters were used for the purpose of comparison. The filters used were mean, median, Gaussian, and 2D convolution. Afterwards, adaptive histogram equalization was applied so that images became clear. A segmentation algorithm was applied for the proper segmentation of images. This step used k-means clustering and fuzzy c-means clustering for segmentation. After segmentation, with the help of GLCM (Gray Level Cooccurrence Matrix), 8 features, i.e., contrast, energy, entropy, homogeneity, sum of entropy, sum of variance, dissimilarity, and sum of average, were extracted from the images to form the dataset of 41 CT scan images (20 were from [34] and 20 were from a different paper: Abnormalities Detection in CT Scan Lung Images Using GLCM [37]) where 28 are lung cancer patients and 13 are patients not affected by cancer. The use of two datasets makes the results more generalized. Ensemble learning was used for the classification of the dataset. Bagging and gradient boosting (a part of ensemble learning) were used for classification.  Figure 1 shows the block diagram of framework for detection of lung cancer.

### 3.1. Filtering

#### 3.1.1. Mean Filter

It blurs the image to reduce noise to a minimum. It involves calculating the mean values of pixels in the *m* × *m* kernel. The mean will replace the intensity of the center element's pixel. This results in smoothing and removal of noise up to a certain extent. This can be implemented using the OpenCV library. For color images, it is necessary to convert the images from RGB to HSV, as the dimensions of RGB are interdependent, and the dimensions of HSV are independent separately.

#### 3.1.2. Gaussian Filter

This filter is similar to the mean filter, but it calculates the weighted mean of the neighboring pixels having a parameter sigma with a discrete approximation. The kernel represents the value of the Gaussian distribution. Although it blurs edges like a standard filter, it is good at protecting edges compared to similar-sized filters. This can also be implemented using the OpenCV package. It allows us to specify the kernel's size.

#### 3.1.3. Median Filter

This filter calculates the median of neighboring pixels to the center in the *m* × *m* kernel. The median then changes the center pixel. It does an excellent job in removing slight noises compared to mean and Gaussian filters. It also preserves the edges of the image but fails to deal with speckle noise. This can also be implemented using the OpenCV library.

#### 3.1.4. 2D Convolution Filter

When applying a 2D Convolution filter, images are filtered utilizing Low Pass Filters (LPF) and High Pass Filters (HPF). Low Pass Filter blurs the image and removes noise. High Pass filters detect edges. For each pixel, a 3 × 3 window is centered on this pixel. All pixels falling within this frame are added, and then, the result is divided by 9. It is equivalent to computing the average pixel value inside that frame. This is performed for all image pixel values to give an output filtered image.

**Figure 2:**
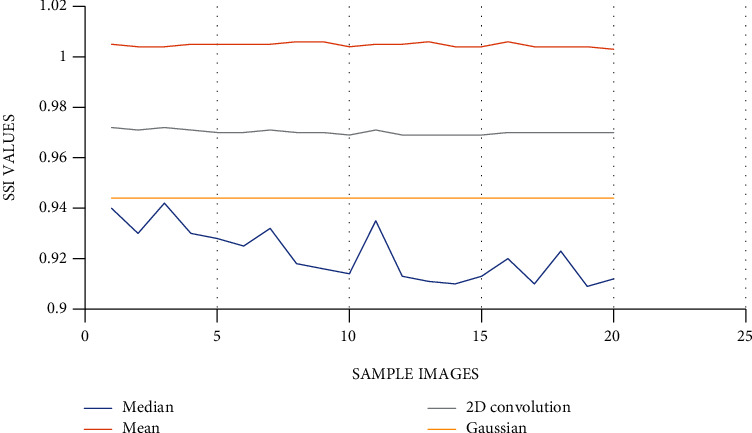
SSI comparison of filters using graph.

**Figure 3:**
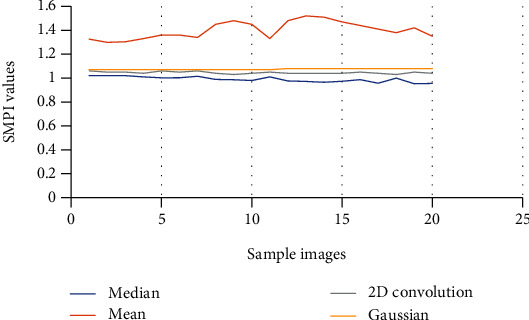
SMPI comparison of filters using graph.

*(1) Performance Measure*. Performance measure of all the four filters, i.e., mean, median, Gaussian, and 2D convolution, is done by comparing SMPI (Speckle Suppression and Mean Preservation Index) and SSI (Speckle Suppression Index) metrics. Per these indices, a lower value represents better performance of filters for mean preservation and noise reduction.  Figure 2 shows the SSI comparison of filters using graph.  Figure 3 gives the SMPI comparison of filters using graph.

(1) $$\begin{array}{c} \text{SSI}=\frac{\sqrt{\text{Variance }\left(\text{final Image}\right)}}{\text{mean }\left(\text{final image}\right)}\times \frac{\text{mean }\left(\text{Initial Image}\right)}{\sqrt{\text{Variance }\left(\text{Initial Image}\right)}},\\ \text{SMPI}=Q\times \frac{\sqrt{\text{Variance }\left(\text{Final Image}\right)}}{\sqrt{\text{Variance }\left(\text{Initial Image}\right)}},\\ Q=1+\left|\text{mean }\left(\text{initial Image}\right)-\text{mean }\left(\text{final Image}\right)\right|. \end{array}$$

In Table 1, the SSI value of the 4 filters (mean, median, Gaussian, and 2D convolution) is provided with their corresponding graphical comparisons in Figure 2. In Table 2, SMPI values of 4 filters are compared, with their graphical comparisons in Figure 3. The lower values of SSI and SMPI denote better preservation of the image after filtering. From the comparison of different filters, as shown in Figures 2 and 3 and Tables 1 and 2, it can be concluded that the median filter is the best and has more accurate characteristics than the remaining filters. Thus, we use median filtered images for image segmentation.

### 3.2. Adaptive Histogram Equalization

**Figure 4:**
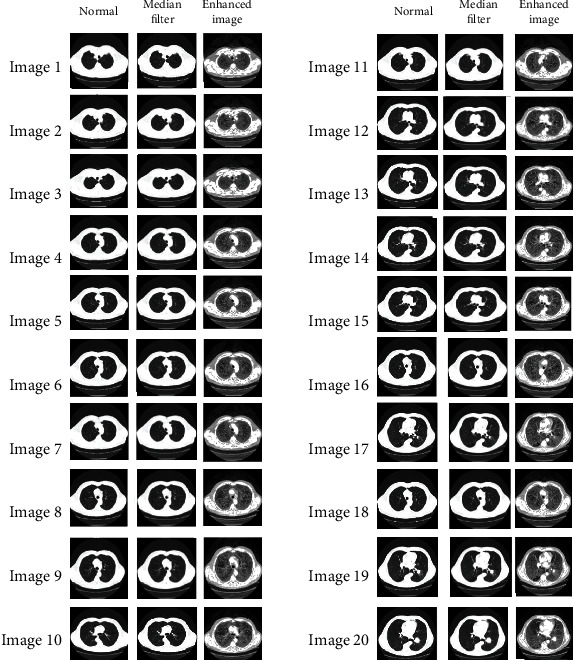
Resultant image (1 to 20) after preprocessing.

The color histogram in image processing addresses the number of pixels in each sort of colored part. Because the histogram equation causes a substantial change in the image's color balance, it cannot be applied independently for an image's red, green, and blue components. However, the algorithm can be applied to the luminance or value channel due to changes in the image's color and saturation if the image is first converted to another color space, such as the HSL/HSV color space. The primary difference between an adaptive histogram and ordinary histogram is that the adaptive approach generates numerous histograms for each image region and utilizes them to redistribute the image's lightness value. Therefore, it is appropriate for refining local contrast in each region of an image and increasing the definition of edges. This step enhances the image, and edges will become sharper and clearer which is necessary for medical image segmentation.  Figure 4 shows the resultant image (1 to 20) after preprocessing.

### 3.3. Image Segmentation

Image segmentation is defined as the method by which a digital image is separated into several different regions, each a set of pixels with distinct objects or similar characteristics. Locating objects and boundaries in images is the main function of image segmentation. It can be divided into several methods. With this strategy, the distinct shapes of cancer cell clusters play an important role in determining how severe the cancer is. In our case, two clustering algorithms were used to perform segmentation of images—k-means clustering and fuzzy-c means clustering.

#### 3.3.1. K-Means Clustering Algorithm

The k-means clustering algorithm is the most basic and classical form of cluster analysis. We apply k-means to separate the given dataset into two or more groups. The method's accuracy is measured by evaluating each cluster center produced by the algorithm, as selecting the proper cluster center is essential for getting the best results. A very simple method to separate the dataset is by using Euclidean distance, which we use to assign pixels to an individual cluster. The following function is used in this algorithm:

(2) $$\begin{array}{c} J=\overset{m}{\underset{i=1}{\sum }}\overset{K}{\underset{k=1}{\sum }}W_{ik}{\left\|x^{i}-\mu_{k}\right\|}^{2}, \end{array}$$

where *x*_*i*_ is the pixels, *v*_*j*_ is the cluster centers, |xi − vj| is the Euclidean distance between *x*_*i*_ and *v*_*j*_, *Ci* is the number of data points for the *i*^th^ cluster, and *C*_*i*_ is the number of cluster centers. Approach k-m to solve the problem is called expectation-maximization. The expectation phase assigns data points to the nearest cluster. The maximization phase calculates the nucleus of each cluster. Below is how we solve it mathematically.

#### 3.3.2. Fuzzy C-Means Clustering Algorithm

Fuzzy clustering (also known as soft clustering or soft k-means) is a clustering method by which each data point can be assigned to multiple clusters. This clustering or cluster analysis includes grouping data points into clusters such that items in the same cluster are as similar as possible, while points in different clusters are as dissimilar as possible. Groups are distinguished through similarity metrics such as distance, connectivity, and intensity. Depending on the data or application, different similarity measures can be employed. The membership of each data point relating to each cluster center is determined by the distance between the cluster center and the data point. The more data in the cluster center, the more membership towards the special cluster center. The membership magnitude of each data point must sum to one, after updating each recursive membership and cluster center principle:

(3) $$\begin{array}{c} \mu_{ij}=\frac{1}{\sum_{k=1}^{c}{\left(d_{ij}/d_{ik}\right)}^{\left(2/m-1\right)}}, \end{array}$$

(4) $$\begin{array}{c} V_{j}=\frac{\left(\sum_{i=1}^{n}{\left(\mu_{ij}\right)}^{m}x_{\text{i}}\right)}{\left(\sum_{i=1}^{n}{\left(\mu_{ij}\right)}^{m}\right),}\forall j=1,2,3..c, \end{array}$$

where

“*μ*_*ij*_” represents the membership of *i*^th^ data to *j*^th^ cluster center. “*c*” represents the number of cluster centers. “*d*_*ij*_” represents the Euclidean distance between *i*^th^ data and *j*^th^ cluster center, and “n” is the number of the data point. “m” is the fuzziness index m € [1, ∞]. “v_j_” represents the *j*^th^ cluster center.

Performance measure: Here, we do the accuracy measure of both clustering algorithms, i.e., k-means and Fuzzy c-means, with a median filter for the segmentation of the image

Accuracy: a performance measure that gives information about the correctness of any process

True positive (TP): foreground pixels are correctly segmented

True negative (TN): background pixels are correctly detected

False positive (FP): foreground pixels are incorrectly segmented

False negative (FN): background pixels are incorrectly detected

**Figure 5:**
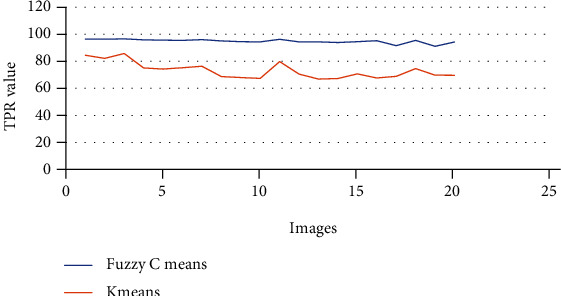
TPR comparison of k-means and fuzzy c-means.

**Figure 6:**
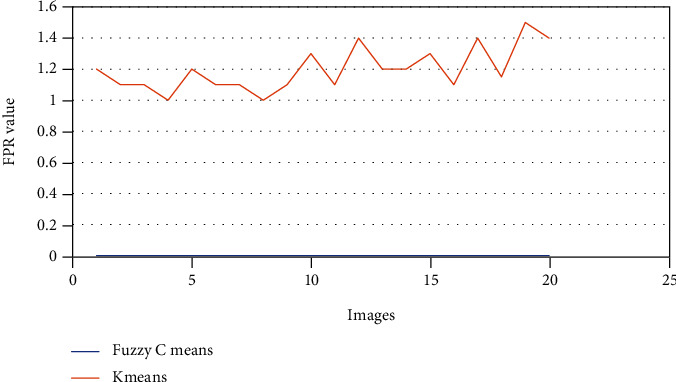
FPR comparison of k-means and fuzzy c-means.

**Figure 7:**
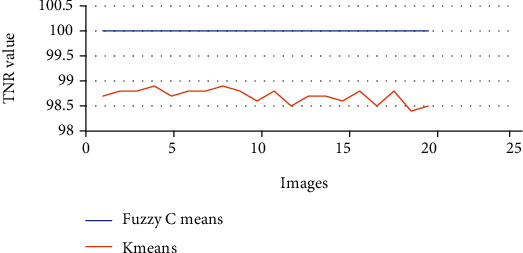
TNR comparison of k-means and fuzzy c-means.

**Figure 8:**
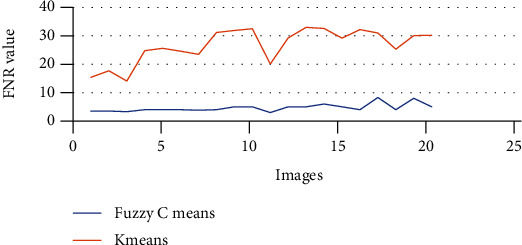
FNR comparison of k-means and fuzzy c-means.

**Figure 9:**
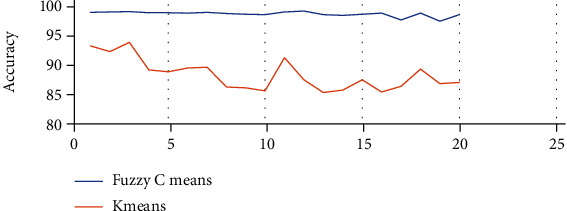
Accuracy comparison of k-means and fuzzy c-means.

The above Tables 3 and 4 show the true positive rate, true negative rate, false positive rate, false negative rate, and accuracy of k-means clustering algorithm (Table 3) and fuzzy c-means clustering algorithm (Table 4).  Figure 5 shows a graphical comparison of TPR between k-means and fuzzy c-means. Similarly, Figure 6 shows an FPR comparison.  Figure 7 shows the TNR comparison.  Figure 8 shows the FNR comparison.  Figure 9 shows the accuracy comparison between k-means and fuzzy c-means using a graph.

Edge detection in an image is a crucial technique for determining the limits of various distinctive objects. It can be implemented by looking for discontinuities in the brightness. Masks can be used for edge detection. Some of them are Laplacian operators, Sobel, and Canny. They are calculated using dissimilarity between adjacent pixels of the image.

### 3.4. Feature Extraction

Feature extractions from a segmented image yield several important properties that are utilized in defining the segmented image's characteristics. The crucial information of the presence of nodules (or lack thereof), which is used to detect or distinguish between malignant and nonmalignant images, can be diagnosed using the extracted features. 8 Haralick features, namely, contrast, energy, entropy, homogeneity, sum of entropy, sum of variance, dissimilarity, and sum, as shown in Table 5, were extracted by finding GLCM (Gray Level Cooccurrence Matrix). These 8 features of the images were used in the analysis in this study.

#### 3.4.1. Gray Level Cooccurrence Matrix (GLCM)

GLCM is an image analysis technique. It is a statistical method for examining the shape of the pixels of an image as a gray-scale matrix, also known as the gray-scale spatial cooccurrence matrix. It is a classification technique, the final step of which is to train the classifier. Its main function is to extract the texture feature from the image. The GLCM function generates a GLCM and then extracts the statistical functions from this matrix with the specified values and spatial relationship of the shape of an image. The gray-coefficient matrix is derived from the gray-scale coefficient matrix. Gray-level cooccurring grids are also called gray-level spatial dependence grids. The gray-cum-matrix is used to generate the GLCM by computation, but *i*, which usually represents gray-level (gray-level probability), is a valuable, horizontal neighbor to *j*. Each part of the GLCM (*i*, *j*) represents the sum of the image element. The figure below shows the gray-scale coherence grid-matrix (GLCM) of the gray-scale image (*i* and *j* = image element).

Haralick Features:

### 3.5. Classification

#### 3.5.1. Ensemble Learning

Ensemble learning is a method for systematically building and combining a large number of machine learning models in tandem to solve a specific problem. By merging different models, machine learning outcomes can be dramatically improved. This method outperforms a single model in terms of prediction accuracy. Here, 5 models are considered for ensemble learning: decision tree classifier, multilayer perceptron classifier, Support Vector Machine, K-nearest neighbor classifier, and logistic regression classifier. For meta outcome evaluation, we use the maximum voting technique to find optimal accuracy among all 5 models.

#### 3.5.2. Bagging

Bagging is a strategy used to boost the accuracy of a machine learning algorithm. The main goal is the creation of multiple different subsets of data from randomly chosen training samples, and then, substitution is done. The decision trees are trained by different subsets of data. This results in a collection of various models, which oftentimes multiplies the power of a model.

Bagging steps are as follows:

- Suppose that the training dataset has n observations and m characteristics. With substitution, one sample is randomly selected from the training dataset
- A subset of L features is chosen randomly, and the best features are used to iterate over the partition node
- The tree becomes the largest
- Repetition of the above steps is carried out n times, and the prediction is built on the sum of predictions by the number of n trees

#### 3.5.3. Boosting

Boosting is used to convert weak learners to strong learners. It is one of the most used algorithms in data science. In this method, learners are sequentially trained with early learners to fit simple models to the data, after which, the data is analyzed to detect the errors. In order to achieve a progressively higher accuracy in each step from the preceding tree, successive trees are fitted. When a hypothesis implies an input, its weight is increased, making the next hypothesis more likely to be categorized correctly. This technique transforms low-performing learners into high-performing models.

Boosting steps are as follows:

- Weak learner W is trained by drawing a random subset of training sample T without replacement from training set P
- In order to train the weak learner W2, a second random training subset P2 is drawn without replacement from the training set, then 50 percent of the earlier incorrect classified/miscall sample is added
- In order to train the third weak learner W3, training samples P are found in training set P3, on which there is a disagreement between W1 and W2
- All the weak learners are mixed through majority voting
- In order to train the weak learner W2 again, a second random training subset T2 is drawn without replacement from the training set and 50 percent of the earlier incorrect classified/miscall sample is added
- W3, the third weak learner, is trained by finding a training sample P in training set T3 where there is a disagreement between W1 and W2
- Weak learners are again mixed through majority voting

#### 3.5.4. Gradient boosting

The gradient boosting machine (GBM) is a machine learning technique for boosting, regression, and classification problems that generates weak prediction models, usually a prediction model combined with a decision tree. It is an ensemble learning method where the weak models used are decision trees. It defines a loss function and minimizes it. It builds step-by-step models just like other boosting methods and simplifies them by allowing optimization of the arbitrary differential loss function. Gradient boosting can be understood more easily with the basic idea of AdaBoost. Gradient boosting is a proven powerful algorithm to build a predictive model, which is why we tested and selected it here.

## 4. Results and Discussions

A confusion matrix is a table that shows how well a classification model (or “classifier”) performs on a set of test data for which the true values are known. This enables the performance of an algorithm to be visualized.

**Figure 10:**
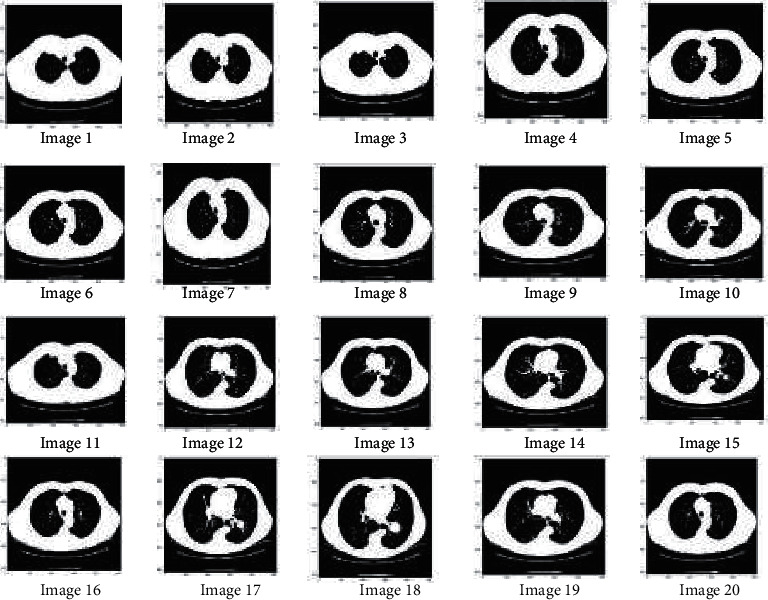
Resultant image (1 to 20) after segmentation.

**Figure 11:**
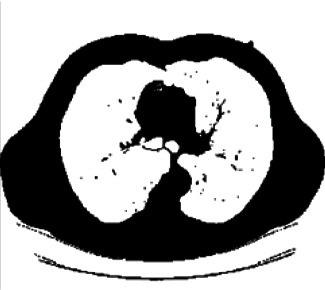
Thresholding.

**Figure 12:**
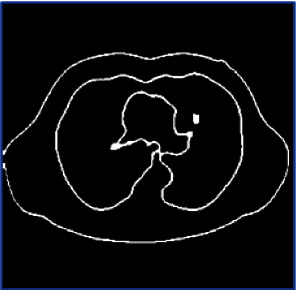
Masking.

**Figure 13:**
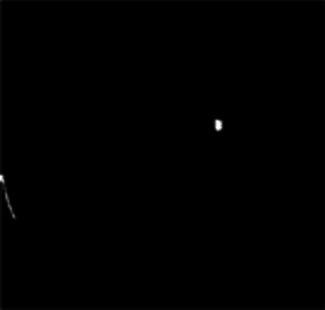
Extraction.

In the preprocessing step, the performance of the median filter was the best among all the other tested filters—mean, Gaussian, and 2D convolution. From the SMPI and SSI values as shown in Tables 1 and 2 and Figures 2 and 3, it can be found that the image segmentation using a median filter has better performance than a mean filter—Gaussian and 2D convolution. True positive rate, true negative rate, false positive rate, and false negative rate were used to calculate the segmentation accuracy. For segmentation, the accuracy of fuzzy c-means clustering is higher than the k-means clustering algorithm. Fuzzy c-means achieves 97% accuracy. All the results are shown in Tables 3 and 4. All the comparisons of TPR, TNR, FNR, and FPR are shown in Figures 5–8. The accuracy comparison between k-means and fuzzy c-means was shown in Figure 9. The results show that the fuzzy c-means clustering algorithm outperforms k-means for lung cancer CT image segmentation. After that, the dataset was obtained by extracting Haralick features of 41 CT scan images (21 were from [34], and 20 were from abnormalities detection in CT scan lung images using GLCM [37]) and was classified using an ensemble learning algorithm. The resultant image of all 20 images after segmentation is shown in Figure 10. The output after thresholding, masking, and extraction is shown in Figures 11–13.

**Figure 14:**
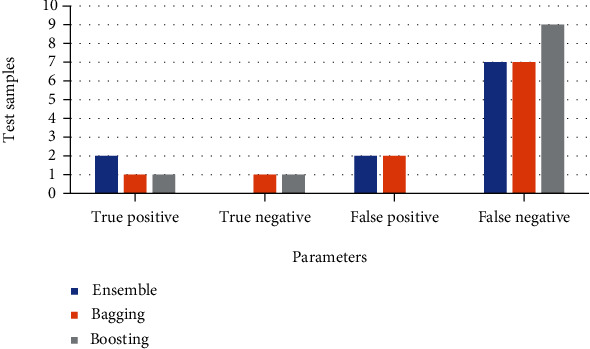
Confusion matrix of various classification algorithms.

**Figure 15:**
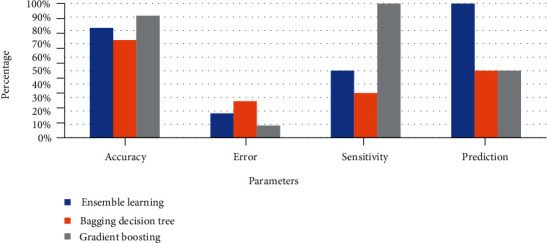
Performance measure of various classification algorithms.

The dataset was trained under 8 features and split into 75% for training the model and 25% for testing the model. The classifiers used in ensemble learning are DT, KNN, MLPNN, SVM, and logistic regression, with bagging using decision tree and gradient boosting. The performance measure of ensemble learning, bagging, and gradient boosting represented through a confusion matrix is shown in Table 6, and classification accuracy is compared in Table 7. The comparison of TP, TN, TP, and FP is shown in Figure 14, and a comparison of accuracy, sensitivity, and specificity is shown in Figure 15. Table 7 shows that the accuracy measure of gradient boosting was 90.9% which was found to be the highest.

A comparison between the proposed study and [34] was performed. The analysis was done using the same dataset.  Table 8 shows that the proposed work achieved a higher accuracy of 98.78% using Fuzzy c-means.

A comparative study between existing and proposed methods is shown below in Table 8.

By combining two datasets [34, 37, 53, 54] into one, the study provided results that could be generalized. The limitation of this study is that the analysis and modeling are not powerful enough for even larger datasets.

## 5. Conclusions

In this paper, we performed image detection for lung cancer by combining the different strategies of GLCM texture extraction and ensemble learning for model-building. The first step, before undertaking any statistical analysis, was preprocessing the medical images. The median filter performed the best as shown by the result's superior SSI and SMPI metric values. Afterwards, clustering was implemented to achieve image segmentation for the cancer specimens. The fuzzy c-map clustering algorithm yielded the best results with a maximum accuracy of 98.78% and accuracy across all images of at least 95%. The classification of cancer was performed by implementing ensemble learning, which is the strategy of aggregating multiple models to reach a more generalized consensus. Developing the model also integrated the techniques of maximum voting, bagging, and gradient boosting. Gradient boosting helped improve the accuracy to 90.9%. Overall, the proposed framework achieved very high performance, with 98.78% accuracy in segmentation and 90.9% accuracy in classification. Thus, this proposed framework can assist medical practitioners and augment modern techniques in medical computer-aided diagnosis of lung cancer.

## Figures and Tables

**Algorithm 1:**
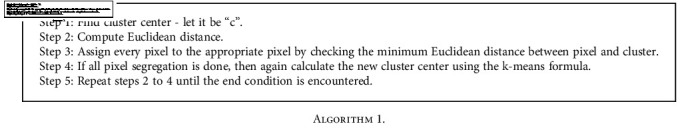


**Algorithm 2:**
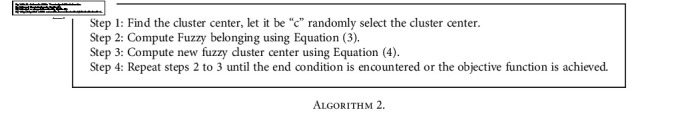


**Table 1:** SSI values of different filters.

Images	Median	Mean	2D convolution	Gaussian
1	0.94	1.005	0.972	0.944
2	0.93	1.004	0.971	0.944
3	0.942	1.004	0.972	0.944
4	0.93	1.005	0.971	0.944
5	0.928	1.005	0.97	0.944
6	0.925	1.005	0.97	0.944
7	0.932	1.005	0.971	0.944
8	0.918	1.006	0.97	0.944
9	0.916	1.006	0.97	0.944
10	0.914	1.004	0.969	0.944
11	0.935	1.005	0.971	0.944
12	0.913	1.005	0.969	0.944
13	0.911	1.006	0.969	0.944
14	0.91	1.004	0.969	0.944
15	0.913	1.004	0.969	0.944
16	0.92	1.006	0.97	0.944
17	0.91	1.004	0.97	0.944
18	0.923	1.004	0.97	0.944
19	0.909	1.004	0.97	0.944
20	0.912	1.003	0.97	0.944

**Table 2:** Comparison of SMPI values of 4 filters.

Images	Median	Mean	2D convolution	Gaussian
1	1.02	1.326	1.06	1.07
2	1.02	1.299	1.05	1.07
3	1.02	1.304	1.05	1.07
4	1.01	1.33	1.04	1.07
5	1.002	1.36	1.06	1.07
6	1.003	1.36	1.05	1.07
7	1.015	1.34	1.06	1.07
8	0.989	1.45	1.04	1.07
9	0.986	1.48	1.03	1.07
10	0.98	1.45	1.04	1.07
11	1.01	1.33	1.05	1.07
12	0.976	1.48	1.04	1.08
13	0.972	1.52	1.04	1.08
14	0.965	1.51	1.04	1.08
15	0.973	1.47	1.04	1.08
16	0.987	1.44	1.05	1.08
17	0.957	1.41	1.04	1.08
18	1	1.38	1.03	1.08
19	0.953	1.42	1.05	1.08
20	0.955	1.35	1.04	1.08

**Table 3:** Performance measure of fuzzy c-means clustering.

Images	TPR	FPR	TNR	FNR	Accuracy
1	96.4	0	100	3.5	98.71
2	96.4	0	100	3.5	98.74
3	96.6	0	100	3.3	98.78
4	95.9	0	100	4	98.63
5	95.7	0	100	4	98.61
6	95.5	0	100	4	98.57
7	96.1	0	100	3.8	98.68
8	95.1	0	100	4	98.51
9	94.6	0	100	5	98.37
10	94.4	0	100	5	98.28
11	96.3	0	100	3	98.74
12	94.4	0	100	5	98.9
13	94.4	0	100	5	98.29
14	93.9	0	100	6	98.15
15	94.6	0	100	5	98.34
16	95.3	0	100	4	98.53
17	91.6	0	100	8.3	97.39
18	95.5	0	100	4	98.56
19	91.1	0	100	8	97.2
20	94.3	0	100	5	98.27

**Table 4:** Performance measure of k-means clustering.

Images	TPR	FPR	TNR	FNR	Accuracy
1	84.5	1.2	98.7	15.4	93.07
2	82.2	1.1	98.8	17.7	92.11
3	85.8	1.1	98.8	14.1	93.65
4	75.1	1	98.9	24.8	89.04
5	74.3	1.2	98.7	25.6	88.71
6	75.3	1.1	98.8	24.6	89.39
7	76.4	1.1	98.8	23.5	89.49
8	68.7	1	98.9	31.2	86.19
9	68	1.1	98.8	31.9	86.02
10	67.4	1.3	98.6	32.5	85.54
11	79.8	1.1	98.8	20.1	91.08
12	70.6	1.4	98.5	29.3	87.35
13	66.9	1.2	98.7	33	85.25
4	67.3	1.2	98.7	32.6	85.65
15	70.7	1.3	98.6	29.2	87.40
16	67.7	1.1	98.8	32.2	85.35
17	68.9	1.4	98.5	31	86.27
18	74.6	1.15	98.8	25.3	89.17
19	69.8	1.5	98.4	30.1	86.71
20	69.7	1.4	98.5	30.2	86.96

**Table 5:** Haralick features extracted from GLCM.

1	Contrast	$\underset{i}{\sum }\underset{j}{\sum }{\left(i-j\right)}^{2}p_{d}\left(i,j\right)$
2	Energy	$Energy=\sqrt{\text{ASM}}$ $\text{ASM}=\underset{i}{\sum }\underset{j}{\sum }{p_{d}}^{2}\left(i,j\right)$
3	Entropy	$-\underset{i}{\sum }\underset{j}{\sum }p_{d}\left(i,j\right)lnln\;p_{d}\left(i,j\right)$
4	Homogeneity	$\underset{i}{\sum }\underset{j}{\sum }\frac{1}{1+{\left(i-j\right)}^{2}}p_{d}\left(i,j\right)$
5	Sum of entropy	$-\overset{2N_{g}}{\underset{i=2}{\sum }}p_{x+y}\left(i\right)\text{loglog }\left\{p_{x+y}\left(i\right)\right\}=f_{8}$
6	Sum of variance	$\overset{2N_{g}}{\underset{i=2}{\sum }}{\left(i-f_{8}\right)}^{2}p_{x+y}\left(i\right)$
7	Dissimilarity	$\overset{N}{\underset{j=1}{\sum }}\left|i-j\right|.p\left(i,j\right)$
8	Sum of average	$\overset{2N_{g}}{\underset{i=2}{\sum }}ip_{x+y}\left(i\right)$

**Table 6:** Confusion matrix of various classification algorithms.

	True positive	True negative	False positive	False negative
Ensemble	2	0	2	7
Bagging	1	1	2	7
Boosting	1	1	0	9

**Table 7:** Comparison of performance measure of various classification algorithms.

	Ensemble learning	Bagging	Boosting
	DT, logistic regression, MLPNN, SVM, KNN	Decision tree	Gradient boosting
Accuracy	81.81%	72.72%	90.90%
Error	18.18%	27.27%	9.09%
Sensitivity	50%	33.33%	100%
Prediction	100%	50%	50%

**Table 8:** 

Paper name	Lung cancer detection using image segmentation by means of various evolutionary algorithms [34]	Lung cancer detection using image processing and classification techniques
Objective	To find a fast image segmentation algorithm for medical images to reduce the time it takes doctors to evaluate computer tomography (CT) scan images.	(i) Classification of lung cancer using extracted Haralick features (ii)Comparing the accuracy of various image segmentation algorithms
Features used	No features used.	Haralick features like contrast, energy, entropy, homogeneity, etc.
Segmentation also used	k-median, -means, particle swarm optimization, guaranteed convergence particle swarm optimization. Inertia-weighted particle swarm optimization, guaranteed convergence particle swarm optimization.	k-means, fuzzy c-means
Results	The highest accuracy is achieved in guaranteed convergence particle swarm optimization, i.e., 95.81%, and the average accuracy is above 90%.	The highest accuracy is achieved in fuzzy c-means, i.e., 98.78%, and the average accuracy is above 95%.

## Data Availability

We can send the datasets at the request of the authors.
